# Focused ultrasound mediated blood–brain barrier opening is safe and feasible in a murine pontine glioma model

**DOI:** 10.1038/s41598-021-85180-y

**Published:** 2021-03-22

**Authors:** Zachary K. Englander, Hong-Jian Wei, Antonios N. Pouliopoulos, Ethan Bendau, Pavan Upadhyayula, Chia-Ing Jan, Eleanora F. Spinazzi, Nina Yoh, Masih Tazhibi, Nicholas M. McQuillan, Tony J. C. Wang, Jeffrey N. Bruce, Peter Canoll, Neil A. Feldstein, Stergios Zacharoulis, Elisa E. Konofagou, Cheng-Chia Wu

**Affiliations:** 1 Department of Neurological Surgery, Columbia University Irving Medical Center, New York, NY 10032 USA; 2 Department of Radiation Oncology, Columbia University Irving Medical Center, 622 W. 168th Street, New York, NY 10032 USA; 3 Department of Biomedical Engineering, Columbia University, New York, NY 10027 USA; 4 Department of Pathology and Cell Biology, Columbia University Irving Medical Center, New York, NY 10032 USA; 5 Division of Molecular Pathology, Department of Pathology, China Medical University and Hospital, Taichung, 40447 Taiwan; 6 Department of Medicine, China Medical University, Taichung, 40447 Taiwan; 7 Translational Cell Therapy Center, Department of Medical Research, China Medical University Hospital, Taichung, 40447 Taiwan; 8 Herbert Irving Comprehensive Cancer Center, New York, NY 10032 USA; 9 Department of Pediatrics, Columbia University Irving Medical Center, New York, NY 10032 USA; 10 Department of Radiology, Columbia University Irving Medical Center, New York, NY 10032 USA

**Keywords:** CNS cancer, Paediatric cancer

## Abstract

Drug delivery in diffuse intrinsic pontine glioma is significantly limited by the blood-brain barrier (BBB). Focused ultrasound (FUS), when combined with the administration of microbubbles can effectively open the BBB permitting the entry of drugs across the cerebrovasculature into the brainstem. Given that the utility of FUS in brainstem malignancies remains unknown, the purpose of our study was to determine the safety and feasibility of this technique in a murine pontine glioma model. A syngeneic orthotopic model was developed by stereotactic injection of PDGF-B^+^PTEN^−/−^p53^−/−^ murine glioma cells into the pons of B6 mice. A single-element, spherical-segment 1.5 MHz ultrasound transducer driven by a function generator through a power amplifier was used with concurrent intravenous microbubble injection for tumor sonication. Mice were randomly assigned to control, FUS and double-FUS groups. Pulse and respiratory rates were continuously monitored during treatment. BBB opening was confirmed with gadolinium-enhanced MRI and Evans blue. Kondziela inverted screen testing and sequential weight lifting measured motor function before and after sonication. A subset of animals were treated with etoposide following ultrasound. Mice were either sacrificed for tissue analysis or serially monitored for survival with daily weights. FUS successfully caused BBB opening while preserving normal cardiorespiratory and motor function. Furthermore, the degree of intra-tumoral hemorrhage and inflammation on H&E in control and treated mice was similar. There was also no difference in weight loss and survival between the groups (*p* > 0.05). Lastly, FUS increased intra-tumoral etoposide concentration by more than fivefold. FUS is a safe and feasible technique for repeated BBB opening and etoposide delivery in a preclinical pontine glioma model.

## Introduction

Diffuse intrinsic pontine glioma (DIPG) is an aggressive pediatric brain tumor with a poor prognosis. These devastating cancers are most commonly diagnosed in children around the ages of 6–7 years, and are responsible for the highest rate of brain tumor-related mortality in the pediatric population^1,2^. The propensity of these tumors to infiltrate within eloquent brainstem tissue precludes surgical resection as a feasible treatment option. Fractionated radiation is the only therapy that provides temporary radiographic and clinical improvement, conferring a survival benefit of 3 months^3^. Nevertheless, local recurrence remains inevitable with most patients demonstrating progression within a year of diagnosis. Median survival in this population is 9 months with only 10% of children alive at 2 years^4,5^.

Over the last several decades, more than 250 clinical trials have studied various combinations and timings of chemotherapeutic agents, though failed to prolong survival. One of the major obstacles limiting the efficacy of systemic therapies in DIPG is the blood–brain barrier (BBB), which prohibits the delivery of most drugs to the central nervous system. To combat this problem, several treatment modalities have been developed to either penetrate or bypass the BBB, including convection-enhanced delivery^6,7^, high-dose chemotherapy^8,9^ and intra-arterial injections^10^. While these are all active areas of study, clinical success has remained limited.

Focused ultrasound (FUS) in combination with intravenously injected microbubbles is an emerging technology that has been shown to cause transient BBB opening without permanent damage^11–13^. In the field of neuro-oncology, FUS has been reported to increase drug delivery and improve survival in preclinical cerebral tumor studies^14–19^, and is currently undergoing early stage testing in humans^20–22^. Furthermore, several recent papers have demonstrated the efficacy of FUS-enhanced drug delivery to the rodent brainstem not harboring tumors^23–25^. However, no study has yet to show FUS-mediated BBB opening in a brainstem tumor.

The main goal of this study was to apply FUS technology to a syngeneic mouse model of pontine high-grade glioma. We hypothesized that FUS-mediated local blood–brain barrier opening in this model was both feasible and safe.

## Materials and methods

### Animal studies

Animal protocol AC-AAAW1464 was reviewed and approved for this study by the internal animal ethics committee, the Columbia University Institutional Animal Care and Use Committee (IACUC), and was in compliance with the ARRIVE guidelines. Six to nine-week-old male B6 (Cg)-Tyrc-2J/J mice (B6-albino) purchased from Jackson Laboratories (Bar Harbor, ME) were used in these experiments (n = 41).

### Cell lines

A high-grade glioma cell line was developed using a PDGF-internal ribosomal entry site (IRES) retrovirus implanted into the cerebral white matter of p53^−/−^ PTEN^−/−^ mice^26^. Tumor cells were then isolated and propagated in adherent conditions on polylysine-coated flasks maintained in culture using a basal media DMEM (Thermo Scientific, Waltham, MA, USA) with 0.5% FBS (Thermo Scientific, Waltham, MA, USA), antibiotic–antimycotic (Thermo Scientific, Waltham, MA, USA), N2 supplement (Thermo Scientific, Waltham, MA, USA), and supplemented with recombinant human PDGF-AA (Peprotech, Rocky Hill, NJ, USA) and FGFb (Peprotech, Rocky Hill, NJ, USA) both to a concentration of 10 ng/ml.

### Intracranial implantation

**Figure 1:**
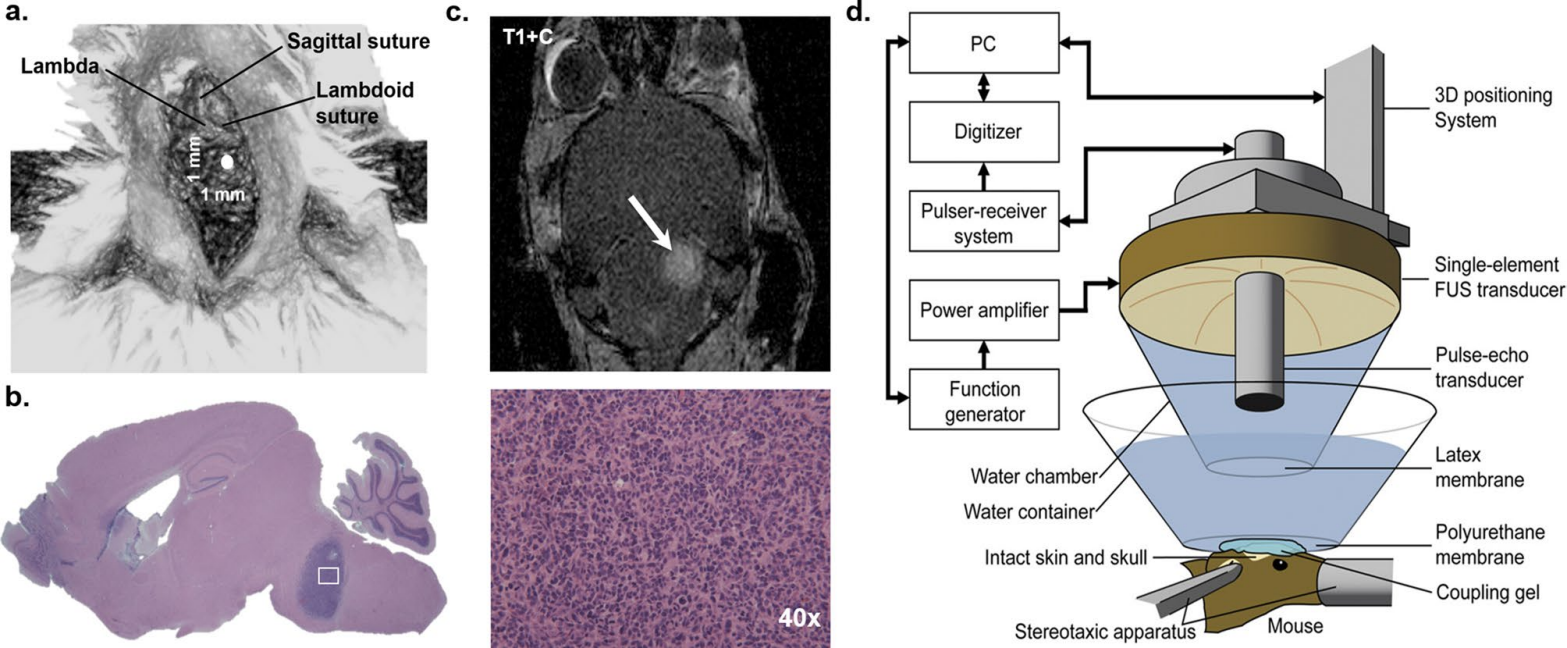
Tumor implantation and experimental setup (**a**) Skull coordinates for intra-pontine tumor implantation. Injections were made 1 mm lateral and 1 mm caudal to the lambda, and 6 mm deep to the skull. (**b**) On H&E, the tumor exhibited hypercellularity and mitotic figures consistent with high-grade glioma. (**c**) Tumor growth was confirmed on contrast-enhanced T1-weighted MR imaging. (**d**) Experimental setup for FUS-mediated BBB opening.

1 × 10^4^ cells were suspended in 1 μl DMEM (Life Technologies, Carlsbad, CA, USA) for orthotopic injection. Before intracranial implantation, mice were anesthetized with a combination of ketamine and xylazine by intraperitoneal injection. The mice were then immobilized in a stereotactic instrument (Stoelting, Wood Dale, IL, USA), a midline 1 cm incision was made in the head, and a hole was drilled in the skull 1 mm posterior to the lambda and 1 mm lateral to the sagittal suture (Fig. 1a). The needle of a Hamilton syringe (Hamilton, Darmstadt, Germany) was introduced to a depth of 6 mm and the cells were injected into the pons over 10 min (Fig. 1b).

### Magnetic resonance imaging

MR imaging for characterization of the size and location of the tumor (Fig. 1c) as well as verification of BBB opening was performed on a 9.4T MRI system (Bruker Medical, Boston, MA, USA). The mice were placed in a birdcage coil (diameter 35 mm), while being anesthetized with 1–2% isoflurane in O_2_ delivered at 0.75/L min. MR images were acquired using a contrast-enhanced T1-weighted 2D FLASH sequence (TR/TE 230/3.3 ms, flip angle: 70°, number of excitations: 4, field of view: 25.6 mm × 25.6 mm, resolution 100 μm × 100 μm × 400 μm) following intraperitoneal bolus injection of 0.2 ml gadodiamide (GD-DTPA) (Omniscan, GE Healthcare, Princeton, NJ, USA), as previously described^27^.

### Experimental design

Ten mice were randomly selected to undergo histological analysis with H&E, five of which were sonicated and five of which were untreated. An additional mouse was treated with ultrasound after Evans blue injection. Another set of 18 mice was randomly chosen for survival analysis: six were used for control, six were treated with one round of ultrasound, and another six were treated twice. The group of six mice that were treated with two courses of FUS also underwent physiological monitoring and sequential weight testing (see below). Lastly, 12 additional mice were used for etoposide delivery experiments. All mice that were sonicated, excluding the Evans dye and etoposide experiments, underwent Kondziela inverted screen testing (n = 17) (see below).

### Focused ultrasound

After MR confirmation of tumor formation, transcranial sonication was performed on day 14. Mice were kept under 1–2% isoflurane anesthesia in 0_2_ delivered at 0.75/L min, and normal body temperature was maintained through a heating pad. Eye drops were applied to prevent drying. A single-element, spherical-segment FUS transducer (center frequency: 1.5 MHz, focal depth: 60 mm, radius: 30 mm; axial full-width half maximum intensity: 7.5 mm, lateral full-width half-maximum intensity: 1 mm, Imasonic, France), driven by a function generator (Agilent, Palo Alto, CA, USA) through a 50-dB power amplifier (E&I, Rochester, NY, USA) was used to target the tumor (Fig. 1d). A central void of the therapeutic transducer held a pulse-echo ultrasound transducer (center frequency: 7.5 MHz, focal depth: 60 mm, radius 11.2 mm; V320-SU, Olympus NDT, Waltham, MA, USA) used for alignment, with their two foci aligned. The − 6 dB bandwidth of this passive cavitation detector was 74.62% (4.33–9.48 MHz). The imaging transducer was driven by a pulser-receiver (Olympus, Waltham, MA, USA) connected to a digitizer (Gage Applied Technologies, Inc., Lachine, QC, Canada). A cone filled with degassed, distilled water was mounted onto the transducer assembly. The transducers were attached to a computer-controlled 3D positioning system (Velmex Inc., Lachine, QC, Canada), as previously described^27^.

Acoustic emissions were passively recorded using the imaging transducer and were analyzed as previously described^28^. Specifically, we calculated the acoustic energy emitted by microbubbles over the entire sonication duration. Furthermore, we performed frequency analysis to identify microbubble response based on spectral features. Finally, we calculated cavitation doses, i.e. stable cavitation doses based on harmonics and ultraharmonics (SCDh and SCDu) and inertial cavitation doses (ICD), as described elsewhere^29^.

To assess for cardiopulmonary effects of ultrasound, an independent experiment of six mice underwent continuous cardiopulmonary monitoring, before, during and after sonication. A Mouse Ox Plus pulse oximeter (Starr Life Sciences Corp, Oakmont, USA) was clamped onto the right hind leg and a Biopac pressure pad with a respiratory transducer (Biopac Systems, Inc., CA, USA) was placed beneath the animal.

Immediately after the injection of microbubbles (SonoVue, Bracco, Milan, Italy or Definity, Lantheus Medical Imaging, North Billerica, MA, USA, which produce similar BBB opening^30^) ultrasound was applied once at each of four points on a 2 mm × 2 mm grid. The sonications were delivered at 1.5 MHz with 0.7 MPa of acoustic pressure in bursts of 10 ms length at 5 Hz repetition time over 120 s (600 pulses). 100 μl of microbubbles were slowly injected prior to the first and third sonication points. To target the tumor, the transducer, which was placed perpendicular to the brain surface, was aligned with the lambda, using a method previously described^31^, and was then adjusted accordingly based on the axial T1-weighted MR image. Follow-up MR imaging was then obtained within two hours to confirm BBB opening and evaluate for acute hemorrhage. A subgroup of mice underwent a second sonication on post-injection day 19.

### Motor and coordination testing

Kondziela inverted screen testing^32,33^ was performed for 60 s immediately prior to sonication to measure strength and coordination. This was then repeated approximately one hour after the mice recovered from inhaled anesthesia. A weights test, as previously described by Deacon^33^, was performed with sequential weight lifting in a subgroup of the mice (n = 6) after undergoing pre-training for two days. While suspended by their tails, mice were taught to grasp onto a steel wool pad connected to a bag of weights. Scoring was calculated by multiplying the quantity of weights and the amount of time suspended. Testing was completed immediately prior to sonication and then on post-treatment day one.

### Survival analysis

Tumor-bearing mice were randomly assigned to control, one-time treatment, or double-treatment groups. The animals were observed and weighed daily following treatment. Primary endpoints of the survival study were weight loss of 20% or higher, signs of severe sickness such as hunched body posture, or death. Log-rank test was used to perform statistical analysis of animal survival, with values of *p* < 0.05 considered significant.

### Histology

To assess for potential injury associated with FUS, a subset of mice implanted with brainstem glioma were divided into two groups, FUS-treated and control. These mice underwent cardiac perfusion within five hours of sonication following confirmation of BBB opening. Their brains were collected and fixed in 4% paraformaldehyde. Hematoxylin and eosin staining was performed and the slides were analyzed by two neuropathologists (PC and CIJ) blinded to the treatment group. The degree of intratumoral hemorrhage and inflammation, as well as intraparenchymal injury was qualitatively analyzed and compared. To demonstrate histological BBB-opening, one mouse underwent intraperitoneal injection of 2% Evans blue dye following sonication, with subsequent cardiac perfusion and brain fixation in 4% paraformaldehyde.

### Liquid chromatography-mass spectroscopy

The last subset of 12 mice were randomly divided into treatment and control groups. Following MR confirmation of tumor growth on post-injection day 14, six mice underwent tumor sonication. Immediately after ultrasound treatment, 20 mg/kg of etoposide was administered via intraperitoneal injection to the treatment group. The remaining control mice were also given intraperitoneal etoposide. The animals were then anesthetized 90 min later with intraperitoneal ketamine and xylazine. Blood samples were collected by cardiac puncture. Transcardial perfusion with 0.9% sodium chloride solution was then performed for 10 min and the brain tumor were isolated. Whole blood samples were allowed to clot for 60 min and then centrifuged at 2000× *g* for 20 min to acquire the supernatants (serum). Both blood and tumor samples were stored at − 80 °C and then analyzed with liquid chromatography with tandem mass spectroscopy (LC–MS/MS) by the Biomarkers Core Laboratory of the Irving Institute for Clinical and Translational Research (Columbia University, New York, NY). Of note, two of the treatment mice expired prematurely during cardiac perfusion and their samples were unable to be processed for LC–MS/MS.

### Ethical approval

All applicable institutional guidelines set by the Institutional Animal Care and Use Committee (IACUC) for the care and use of animals were followed. The study was carried out in compliance with the ARRIVE guidelines.

## Results

### BBB opening confirmation

**Figure 2:**
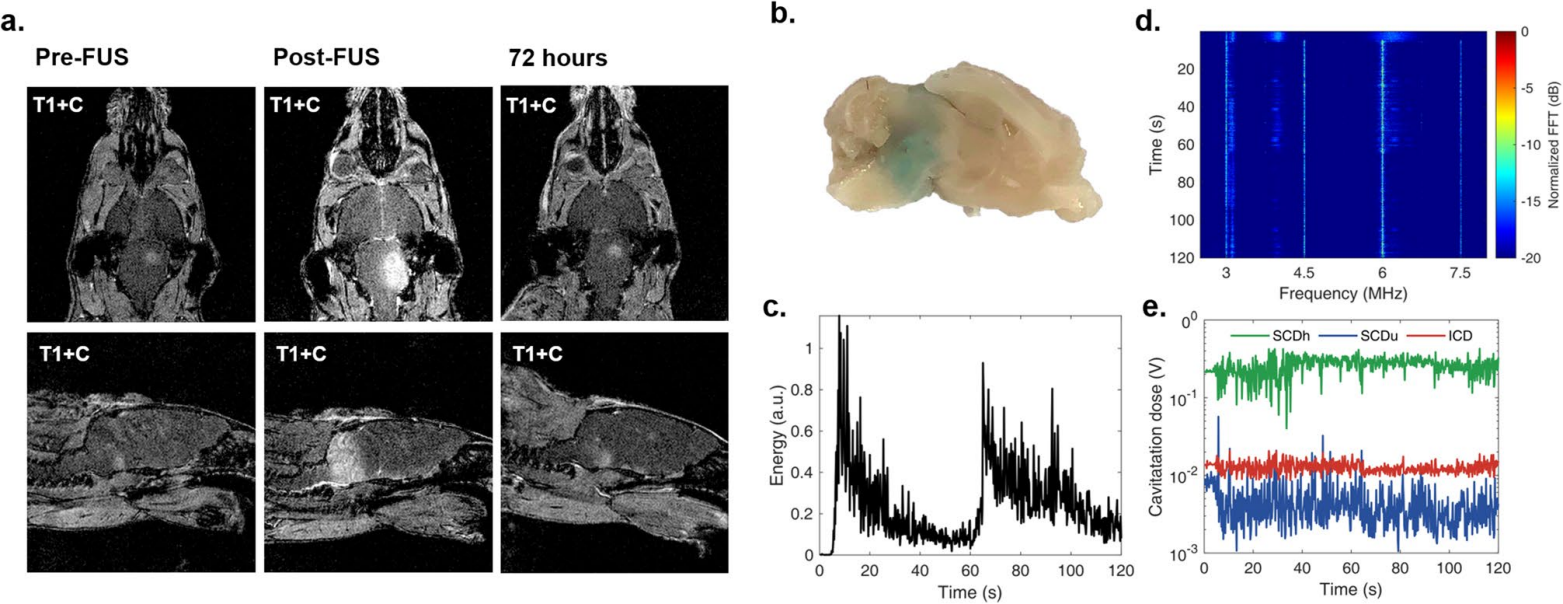
BBB opening and recorded acoustic emissions with passive cavitation measurements. (**a**) T1-weighted MR imaging showing sonication of tumor and peritumoral region. BBB closure demonstrated at 72 h after FUS. (**b**) Evans blue staining demonstrates BBB disruption on gross histology. (**c**) In vivo passive cavitation detection measurements confirmed that stable cavitation dominated throughout ultrasound treatment. Acoustic energy emitted by the microbubbles was higher in the beginning of the treatment, and gradually decreased due to microbubble clearance from the vasculature. (**d**) Spectrogram of the entire treatment session. Higher harmonic emissions were detected, with no substantial increase in the broadband floor after microbubble entrance into the focal volume. (**e**) Stable harmonic cavitation levels rose slightly after microbubble administration at 0 and 60 s and remained relatively constant throughout the sonication. Stable ultraharmonic (blue line) and inertial cavitation levels (red line) showed an absence of violent cavitation events. FFT = fast Fourier transform.

All animals underwent MR imaging on day 13 post injection, and those with pontine tumors were selected for the study (Figs. 1c, 2a). Acoustic energy emitted by the microbubbles was higher in the beginning of the treatment, and gradually decreased due to microbubble clearance from the vasculature. Stable cavitation emissions dominated the spectra during the FUS treatment. Passive cavitation detection measurements of one mouse are shown in Fig. 2c–e. Energy increase was observed after the second microbubble injection, on the third treated site. SCDh was an order of magnitude higher than ICD and two orders of magnitude higher than SCDu, verifying that stable microbubble activity drove the BBB opening process. Localized BBB opening was confirmed with contrast-enhanced T1-weighted imaging in all mice that underwent sonication. Evans blue injection performed in one of these mice revealed gross histological BBB-opening, which mirrored the pattern of enhancement seen on MR imaging (Fig. 2b). BBB closing was observed 72 h after sonication. (Fig. 2a).

### Physiological monitoring during BBB opening

**Figure 3:**
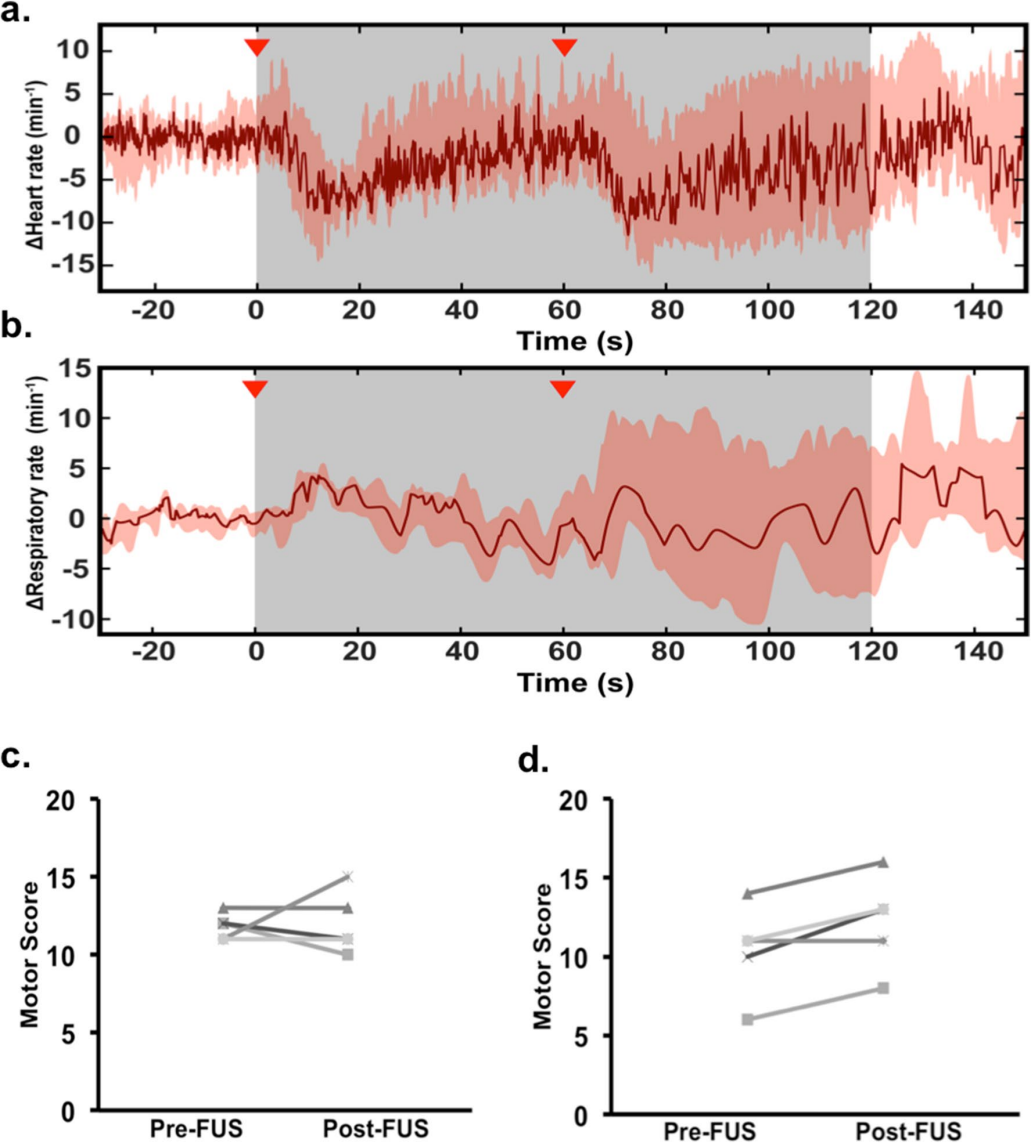
Monitoring of cardiopulmonary vitals and motor testing. (**a**) The grey region indicates the period of sonication. Mean change in pulse across all mice showed injection-associated (red arrows) physiological dip in heart rate that spontaneously resolves. No cardiac pause or apnea was observed. (**b**) Mean respiratory rate recording showed no significant fluctuations or change. Shaded areas represent one standard deviation. (**c**) Weights test in FUS group revealed no difference in motor score before and after FUS (*p* = 0.8470). (**d**) After the second FUS, mice did show a significant trend with improved motor testing (*p* = 0.0301).

After anesthesia induction, in a subset of mice (n = 6), heart rate and respiratory rate were recorded for 90 s prior to sonication to establish baseline measurements. During sonication, there was no observed pathophysiological response including cardiac pause or apnea. Mean change in heart rate and respiratory rate with standard deviation across all six mice are represented in Fig. 3a,b. FUS did not lead to a significant change in vitals from pre-treatment measurements. We did observe a physiological drop in heart rate immediately after intravenous microbubble injection, which returned back to baseline.

### Motor and coordination testing before and after sonication

In all mice that underwent sonication (n = 17), the procedure was well tolerated and there was no observed difference in behavior or development of new symptoms. Furthermore, all mice successfully completed the 60-s Kondziela inverted screen test both before and after treatment without any changes (data not shown). The sequential weights test revealed no significant change in motor score during the first FUS treatment (*p* = 0.8494), but improved scores after the second treatment (*p* = 0.0301) (Fig. 3c,d).

### Weight monitoring and survival analysis

**Figure 4:**
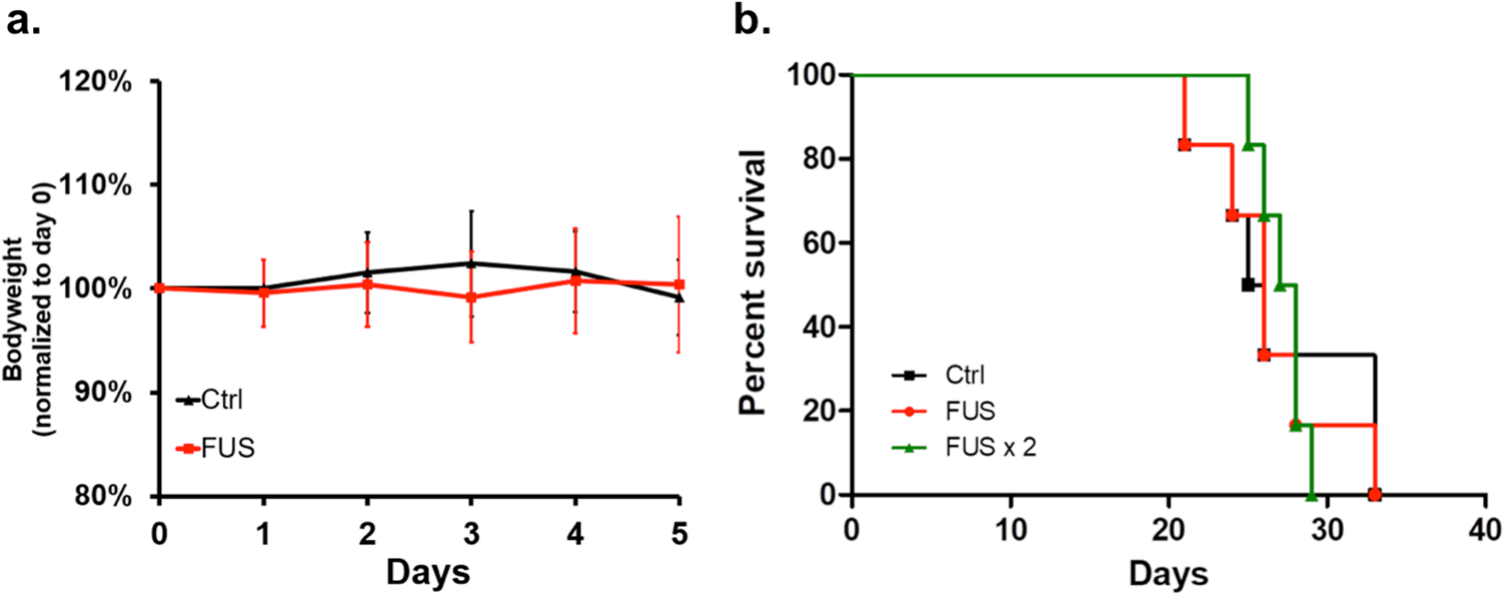
(**a**) Bodyweight of control animals and FUS-treated animals during the five-day post-treatment period. No significant weight loss was observed. (**b**) Kaplan–Meier curve reveals no significant difference between the survival of control mice (n = 6) with FUS (n = 6) (*p* = 0.8037), and double-FUS treated mice (n = 6) (*P* = 0.9399).

To assess safety and feasibility of repeated FUS-mediated BBB-opening, mice harboring brainstem glioma underwent single FUS treatment or two FUS treatments one week apart allowing adequate BBB-closure. One mouse died as injection began which was likely related to over sedation or air bubbles in the catheter, and was not included in the analysis. Across both the FUS and double-FUS groups, mice did not show any significant decline in weight in the first five days after treatment (Fig. 4a). Log Rank analysis demonstrated statistically similar survival times between the control group (n = 6, mean survival = 27.0 days), the FUS group (n = 6, mean survival = 26.3 days, *p* = 0.8037), and the double-treatment group (n = 6, mean survival = 27.2 days, *p* = 0.9399) (Fig. 4b).

### Histological analysis

**Figure 5:**
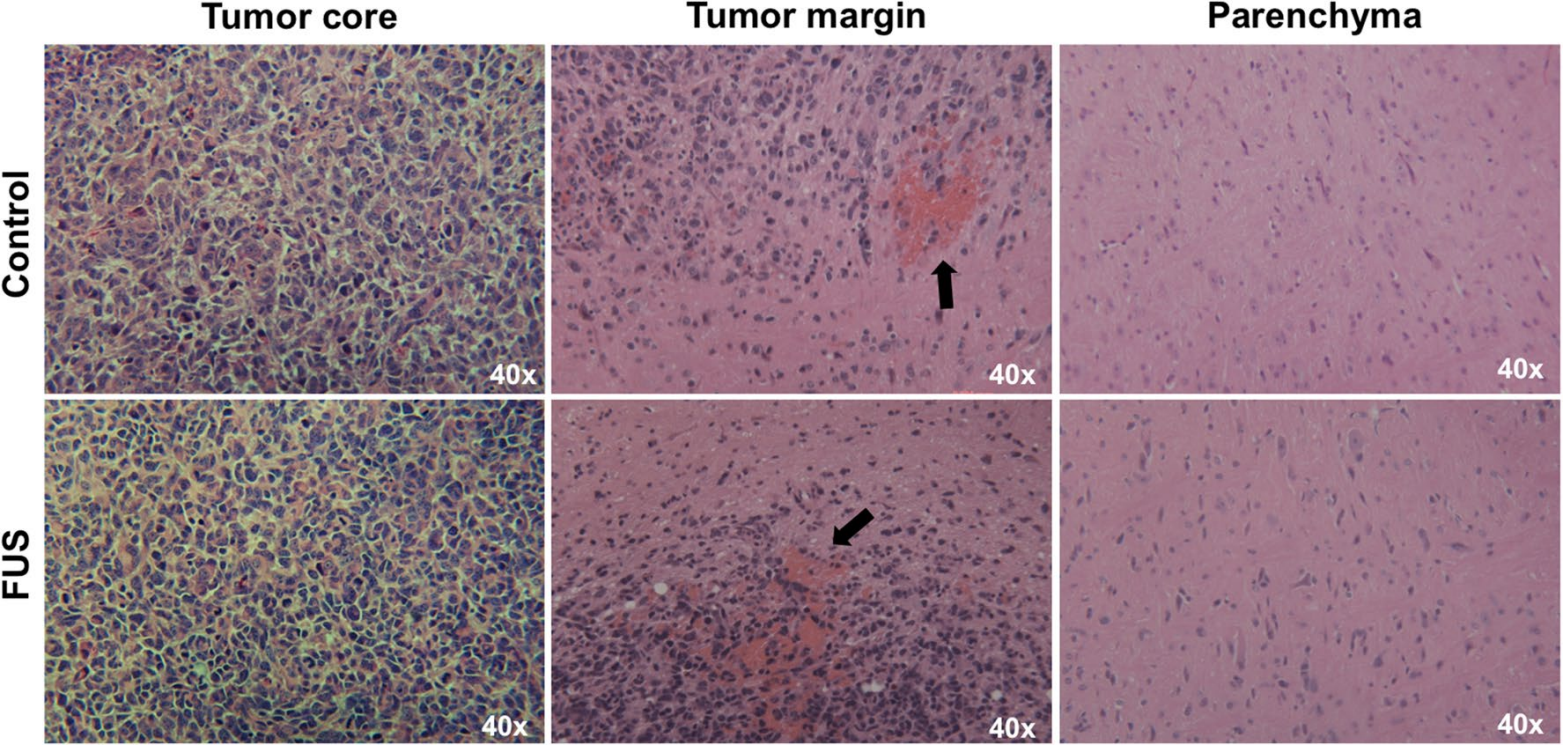
Representative photomicrographs of H&E-stained tissue from control and treated mice. Review of the histology by two blinded neuropathologists showed no difference. Tumor margins in both groups showed regions of microhemorrhage (black arrows) likely secondary to tumor invasion. No peri-tumoral parenchymal injury was seen.

To assess for histological evidence of injury from ultrasound, a separate set of experiments were performed. Mice implanted with brainstem glioma were separated into two groups, FUS-treated (treated) or control group. Two blinded neuropathologists (PC and CIJ) reviewed H&E slides from treated (n = 5) and control (n = 5) mice and did not differentiate tissue section slides based on treatment group. All samples showed features of high-grade glioma with increased cellularity and proliferation. However, there was no difference in abundance of hemorrhage or degree of inflammation (Fig. 5). Lastly, there was no evidence of parenchymal injury or necrosis.

### Etoposide delivery

**Figure 6:**
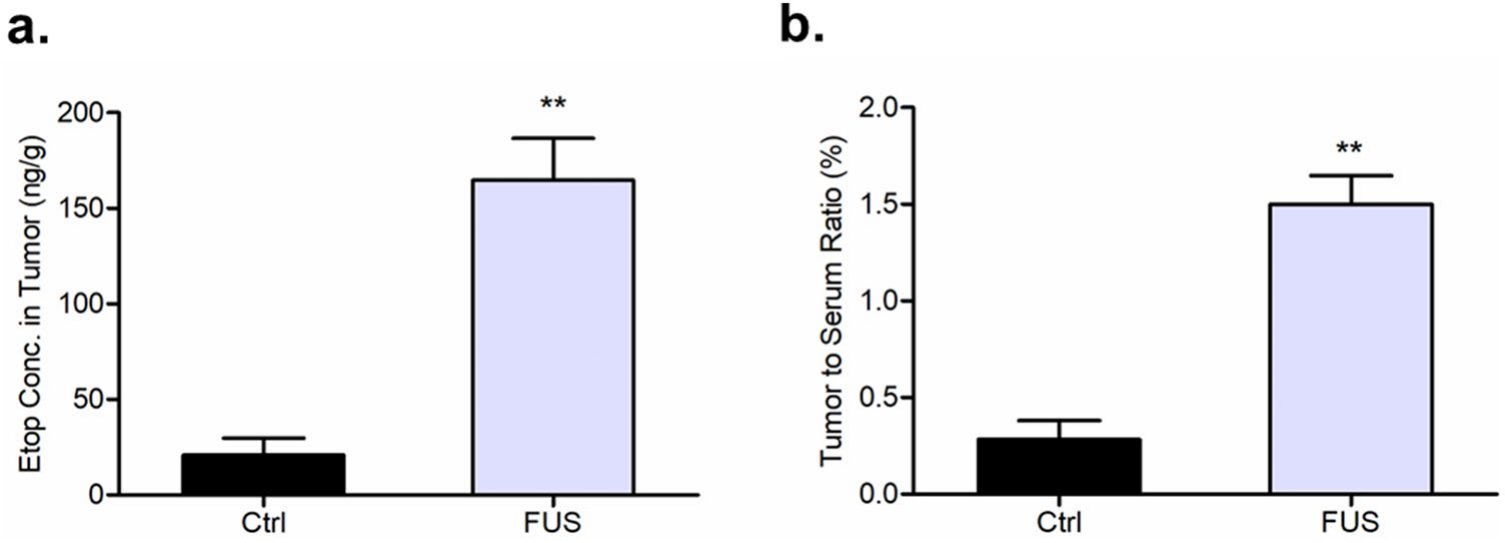
LC–MS/MS measurements of etoposide delivery. (**a**) Concentration of etoposide in brain tumors with and without sonication. (**b**) The tumor-to-serum ratios of etoposide concentration is more than fivefold greater in the treatment group (*p* < 0.005).

LC–MS/MS was used to demonstrate the ability of FUS to augment the delivery of etoposide in our pontine tumor model. Compared to the tumor tissue in the control group (mean = 20.98 ng/g), the concentration of etoposide in the sonicated tumor tissue (mean = 164.77 ng/g) was almost eight times greater. (Fig. 6a). Furthermore, the mean brain tumor–to-serum ratio was more than fivefold increased in the sonicated mice (1.50%) compared with the non-sonicated mice (0.28%) (*p* < 0.005) (Fig. 6b).

## Discussion

Despite decades of research and clinical trials, DIPG still carries a mean survival of nine months^4^. The diffuse nature of the tumor and eloquence of the brainstem limits surgical resection. While a better understanding of the molecular characteristics of these tumors has allowed for the development of targeted therapies, the BBB remains a significant obstacle for delivering appropriate drug concentrations to the tumor. Furthermore, recent preclinical work suggests that drug delivery to the brainstem may be more challenging than in other regions of the central nervous system^34^. Several rodent studies have demonstrated reduced BBB permeability^35^ as well as a lower density of capillaries^36^ in the brainstem. In light of this, numerous strategies, both surgical and non-invasive, have been developed and trialed over the years to optimize intratumoral drug concentrations.

High dose chemotherapy can cause life-threatening toxicity and has yet to show any effect on survival^8,9^. Mannitol, a hyperosmotic agent, and other drugs such as bradykinin agonists, can non-invasively increase BBB permeability^37^. However, it performs this in a non-selective manner, and can cause global CNS toxicity. Another option under investigation is intra-arterial (IA) drug delivery. IA therapy utilizes selective injection into arterial sources of the tumor, enabling higher concentrations of drugs to be delivered to the tumor bed before it gets dispersed in the systemic circulation. However, this method of drug delivery carries risks of hemorrhage and embolism. Furthermore, it has also been associated with a streaming phenomenon—an uneven distribution of drugs in the posterior circulation that can cause heterogeneous drug distribution with dangerously high concentration of therapy in some regions and limited concentrations in other regions^38^. Convection enhanced delivery (CED) involves the direct infusion of drug to a targeted area of the brain via an infusion catheter^6,7^. Some of the challenges with this strategy lie in the invasive nature of the treatment as well as the high interstitial pressures of the tumor that limit the diffusion of drugs.

FUS is a non-invasive technique that can transiently open the BBB and is safe for repeated procedures. Furthermore, the region that is being treated can be targeted with millimeter accuracy, which is crucial when treating regions of critical importance such as the pons. Many groups have successfully leveraged this technology in preclinical cerebral tumor studies, showing a benefit in both slowing tumor growth and increasing survival^14–19^. There are currently ongoing clinical trials evaluating safety and efficacy of FUS-mediated drug delivery in supratentorial tumors; however, there is a paucity of understanding on the feasibility and safety of FUS-mediated BBB-opening in the brainstem for brainstem gliomas. In the last two years, several groups began to study the efficacy of BBB-opening in the brainstem. Ye et al. demonstrated the feasibility of FUS-enhanced delivery of both intravenously and intranasally administered ^64^Cu-integrated gold nanoclusters to the pons^24^. Additionally, Alli et al. showed that BBB opening in the brainstem of rodents does not alter normal cardiopulmonary or motor function, and preserves hindbrain tissue integrity^25^. Furthermore, they successfully showed elevated brainstem concentrations of doxorubicin in rodents that underwent FUS-mediated BBB-opening. These studies were performed in mice with intact brainstems without the presence of diffuse glioma. Gliomas are diffusely infiltrative tumors with abnormal tumor vasculature. Furthermore, the brainstem controls many important functions necessary for survival, including heart rate, respiration, and swallowing mechanism. In addition, nerve fibers for motor function travel along the brainstem. Manipulation of the brainstem may be fatal and it is unclear whether the presence of tumor in the brainstem may affect the safety and feasibility of BBB opening. In light of these initial findings, our goal was to translate this technique to a syngeneic rodent brainstem glioma model.

In this study, using a PDGFB-driven high-grade syngeneic pontine glioma model, we evaluated the radiographic, physiological and histological consequences of BBB-opening with FUS. Previous studies have shown FUS can induce an inflammatory response with immune cell infiltration. In order to assess the safety of FUS-mediated BBB opening in a brainstem glioma model, we used an established mouse syngeneic diffuse glioma model^26^ and stereotactically injected the tumors in the pons. Given that the brainstem contains the nuclei and tracts that regulate normal cardiopulmonary function, we chose to monitor vitals during sonication. We did not observe any evidence of cardiac or respiratory deficits secondary to ultrasound treatment. Though, we did see a physiological dip in heart rate immediately after microbubble injection. This effect was most likely related to the large volume injection, as the heart rate quickly returned back to the pre-sonication baseline after 10–20 s. Furthermore, this was likely not observed by Alli et al. given their significantly smaller volume of injected microbubble solution. BBB opening was then confirmed with both gadolinium-enhanced T1-weighted MR imaging and Evans blue dye. Furthermore, T1-post contrast MR images at 72-h showed BBB-closure. Motor and coordination testing remained statistically unchanged before and after sonication, and the sonicated mice survived as long as the control group. For the mice that underwent histological analysis, there was no difference between treated and untreated mice, both within the tumor and the peritumoral brainstem parenchyma.

Our study was also the first to show the safety of repeated BBB openings in the brainstem. Six mice were randomized to undergo a second session of FUS on post-injection day 19. These animals showed successful reopening on post-treatment contrast-enhanced MRI. Furthermore, similar to the findings of Alli et al., these mice actually received better motor scores after their second treatment. This was likely secondary to increasing their testing experience and less likely a neuromodulatory effect. We also found that the survival of mice that underwent multiple ultrasound treatments was similar to the other two groups demonstrating the safety and feasibility of repeated BBB-openings.

Lastly, we successfully demonstrated augmented delivery of etoposide, a chemotherapeutic agent commonly used in pediatric neuro-oncology, in our pontine glioma model. Controlling for differences in administration and uptake with a tumor-to-serum ratio, we found that animals that underwent FUS had more than fivefold increase in etoposide concentration.

While our findings are encouraging, there were a few limitations within our study. First, our tumor cell line is more genetically similar to a proneural glioblastoma and was generated by isolating a cell line derived from a virally induced diffuse glioma model in mice^26^. Historically, the diagnosis of DIPG was made by clinical presentation and radiographic findings; however, with the use of brainstem biopsies, it was found that 70–85% of DIPG possess mutation in histone H3 resulting in H3K27M substitution^39^. This led to the recent WHO classification of central nervous system tumors to define a new entity termed diffuse midline glioma (DMG), which encompasses gliomas that harbor H3K27M mutation. DMG make up a majority of what was classically known as DIPG^40^. This cell line does not harbor the classic mutations of DMG, including the most characteristic H3K27M mutation. Further investigation into this is currently ongoing for targeted drug delivery with FUS, and future in vivo therapeutic studies will need to utilize a more clinically relevant model. Additionally, we used a single-element transducer mounted on a 3D positioning system. While the FUS parameters allow for transcranial sonication, our current small animal setup does not permit pure targeting of tumor without including normal brain tissue within the ultrasound beam, especially for at the early stage of the disease. This issue is mitigated in large animals and humans with the use of neuronavigation technology^29^.

In conclusion, FUS is hereby demonstrated to be a safe and feasible technique to open the BBB and enhance drug delivery in a mouse pontine glioma model including in repeated applications. Although these findings are incremental to the field of FUS, in the setting of brainstem glioma, this is a critical next step for clinical translation of this technology for patients. Future studies will assess efficacy of FUS-mediated drug delivery to most efficiently treat brainstem tumors.
